# Inter-rater and test-retest reliability of the “standardized endoscopic swallowing evaluation for tracheostomy decannulation in critically ill neurologic patients”

**DOI:** 10.1186/s42466-020-00055-3

**Published:** 2020-03-30

**Authors:** Tobias Warnecke, Paul Muhle, Inga Claus, Jens B. Schröder, Bendix Labeit, Sriramya Lapa, Sonja Suntrup-Krueger, Rainer Dziewas

**Affiliations:** 1 Department of Neurology with Institute of Translational Neurology, University Hospital Muenster, Albert-Schweitzer-Campus 1 A, 48149 Muenster, Germany; 2 Institute for Biomagnetism and Biosignalanalysis, University Hospital Muenster, Malmedyweg 15, 48149 Muenster, Germany; 3 Department of Neurology, University Hospital Frankfurt, Theodor-Stern-Kai 7, 60590 Frankfurt/Main, Germany

**Keywords:** Tracheostomy, Decannulation, Intensive care, FEES, Swallowing, Dysphagia

## Abstract

**Background:**

Removal of a tracheostomy tube in critically ill neurologic patients is a difficult issue, particularly due to the high incidence of oropharyngeal dysphagia. For an objective evaluation of decannulation readiness the “Standardized Endoscopic Swallowing Evaluation for Tracheostomy Decannulation in Critically Ill Neurologic Patients” (SESETD) – a stepwise evaluation of ‘secretion management’, ‘spontaneous swallows’ and ‘laryngeal sensibility/cough’ – has been introduced. With the recent study detailed data on inter-rater and test-retest reliability are presented.

**Methods:**

To obtain inter-rater reliability levels both in a group of raters with at least 5 years of experience (‘experts’) and in a group of raters with no or only minor experience using the SESETD (‘non-experts’), for each single item of the protocol and the sum score α-, respectively κ-levels were determined. The ‘experts’ assessed the same videos after a four-week interval to determine test-retest reliability. Ten videos from tracheostomized neurological patients completely weaned from mechanical ventilation were assessed independently by six ‘experts’. 27 ‘non-experts’ applied the SESETD on 5 videos from the same patient population after introduction to the protocol in a one-hour workshop.

**Results:**

For the items ‘secretion management’ and ‘spontaneous swallows’ α-levels were identified at > 0.800 both in the group of ‘experts’ and ‘non-experts’. With regard to the item ‘laryngeal sensibility/cough’ in both groups, the α-level was ≥0.667. With κ-levels of 1.0 for ‘secretion management’, 0.93 for ‘spontaneous swallows’ and 0.76 for ‘laryngeal sensibility/cough’ test-retest reliability showed at least substantial agreement for each item. Intraclass correlation coefficient for the sum score was excellent in both groups (α ≥ 0.90).

**Conclusions:**

The SESETD demonstrates good to excellent agreement for each single item included as well as the sum score in experienced and unexperienced raters supporting its usefulness for implementation in daily clinical routine and as an outcome measure for clinical trials.

## Background

Tracheostomy is a frequently performed procedure on the intensive care unit (ICU) to prevent laryngeal and tracheal damage, to shorten the duration of mechanical ventilation and to reduce the length of stay on the ICU [9, 34]. Removal of a tracheostomy tube is a critical issue during intensive care, particularly when taking care of neurological intensive care patients. The latter are particularly prone to suffer from oropharyngeal dysphagia (OD) which is closely intertwined with aspiration and subsequent respiratory complications. Multiple reasons for OD were identified in this patient collective, such as central lesions that cause a disruption of the widely distributed swallowing network, muscle weakness, reduced consciousness and/or impaired pharyngeal sensory feedback [27, 29, 41, 44]. Dysphagia is a known risk factor for decannulation failure and can be found in up to 70% of tracheostomized patients on the ICU [17]. In stroke patients, severe dysphagia and associated insufficient airway protection are the main reasons for delayed decannulation and patients needing to remain tracheostomized [5].

**Fig. 1:**
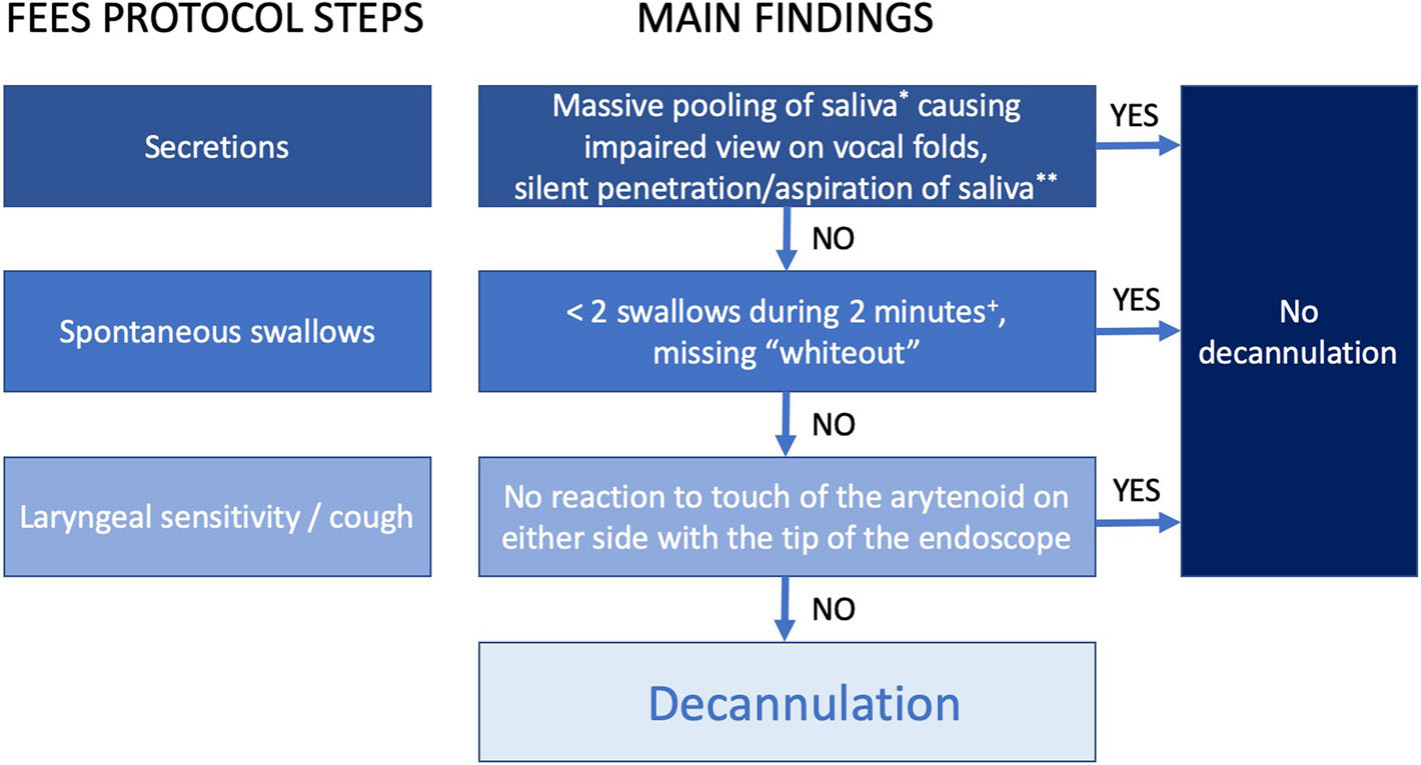
Stepwise evaluation of swallowing function according to the “Standardized endoscopic Swallowing Evaluation for tracheostomy decannulation in critically ill neurologic patients”; *not only coating; **permanently without any reaction; ^+^if exactly two swallows occur in this period, another two minutes of observation are recommended; (adapted from [6]: Warnecke T, Suntrup S, Teismann IK, Hamacher C, Oelenberg S, Dziewas R; Critical Care Medicine 2013 Jul; 41(7):1728–32)

Usually, the decision to decannulate depends strongly on the individual experience of the treating physician [40] and is frequently based on poorly validated or less reliable bedside tools like the modified Evans-blue dye test [4]. To overcome these shortcomings the “**S**tandardized **E**ndoscopic **S**wallowing **E**valuation for **T**racheostomy **D**ecannulation in Critically Ill Neurologic Patients” (further referred to as ‘SESETD’) [44] has been developed and was identified as the only standardized objective tool to evaluate decannulation readiness in a recent systematic review by Singh et al. [39]. The protocol includes the stepwise evaluation of secretion management, spontaneous swallows, sensitivity by touching the arytenoids, swallowing puree and swallowing water during flexible endoscopic evaluation of swallowing (FEES) at bedside (Fig. 1). In the original publication, application of the SESETD-protocol not only showed to be safe evidenced by a decannulation failure rate of only 1.9% but also allowed for significantly more patients to be decannulated than by relying on the clinical swallowing examination alone [28]. The protocol has been implemented in the guidelines of the French Intensive Care Society and the French Society of Anaesthesia and Intensive Care Medicine at a GRADE 2+ recommendation [42] and has been used as a tool to assess the primary endpoint in a recent interventional trial on tracheostomized stroke patients [12, 13].

After having focused on the clinical impact of the SESETD-protocol previously [44], we aimed at providing data on the reliability of the SESETD-protocol in the present study.

## Methods

The **SESETD** consists of a stepwise evaluation of the three items ‘secretion management’, ‘spontaneous swallows’ and ‘laryngeal sensibility/cough’ [44] (Fig. 1). Only if all three items are assessed as passed, patients are considered ready for decannulation. Failure criteria for the item ‘saliva management’ are massive pooling (not only coating) causing an impaired view on the vocal folds and/or silent penetration and/or aspiration of pooled saliva (permanently without any reaction). The item ‘spontaneous swallows’ is considered failed if less than two swallows occurred during 2 minutes of observation. If exactly two swallows occur in this time period, another 2 minutes of observation are recommended. If no reaction to touch of the arytenoid with the tip of the endoscope on both sides could be elicited, the item laryngeal sensibility is considered as failed. The original version of this score also included the two subsequent steps of puree and liquid swallowing [44]. However, since, as stated in the respective publication, all patients who passed the first three items also successfully managed the additional two swallowing tasks, these were discarded thereafter, and, for example, also not used in the multicenter PHAST-TRAC trial [12, 13].

All **videos** used in this study were acquired at the neurological ICU at Münster University Hospital in Germany from tracheostomized and completely weaned patients suffering from cerebral infarction (6/10), intracerebral hemorrhage (2/10), Guillain-Barré syndrome (1/10), and meningoencephalitis (1/10). Passing all three items, 5/10 patients from this collective were ready for decannulation according to the SESEDT. 3/10 patients could not be decannulated because the items ‘spontaneous swallows’ and ‘laryngeal sensibility/cough’ were not passed. 2/10 could not be decannulated due to failing the items ‘secretion management’ and ‘laryngeal sensibility/cough’. During all endoscopic assessments the cuff of the tracheostomy tube was deflated allowing patients to breathe through the upper airway. Videos were recorded using a 3.1-mm-diameter flexible fiberoptic rhinolaryngoscope (11,101 RP2, Karl Storz, Tuttlingen, Germany), a combined light source and camera system delivering videos in standard definition quality (rp CAM-X, rpSzene®, Rehder/Partner, Hamburg, Germany) and a Medical Panel PC (WMP-226, Wincomm Corporation, Hsinchu, Taiwan) that is used for display and recording of the videos. The entire system was contained on a portable instrument cart. Videos were stored in avi-format and sound-track was removed for further analysis. All patients were examined at bedside by an experienced neurologist and a speech-language pathologist (SLT). The videos included endoscopic view only (without additional external view) and were completely anonymized. Every rater filled out a prepared sheet for the items of each assessment. These data were anonymized and then transferred into Microsoft Excel© for further analysis. Data acquisition and analysis were approved by the ethic committee of the “Ärztekammer Westfalen-Lippe and Westfalian Wilhelm University of Münster” (AZ 2019–496-f-S).

**Raters** were separated into two groups depending on their experience using the SESETD: The **‘expert group’** consisted of two neurologists and four SLTs who all had experience using the SESETD-protocol for at least 5 years and were in possession of the FEES instructor certificate [10, 11]. Twenty seven raters (16 physicians and 11 SLTs) with no experience using the SESETD acted as a group of **‘non-experts**’. 10 of these raters had more than 5 years of experience using FEES, 2 raters 3–5 years, respectively 2–3 years, 4 raters had 1–2 years of experience and 9 raters had approximately 1 year of experience using FEES.

The ‘expert group’ rated the ten videos independently from one another twice in randomized order 4 weeks apart. The data from the first evaluation were used to evaluate inter-rater reliability. To assess test-retest reliability, both individual ratings were compared. Raters from the ‘non-expert’-group were introduced to the protocol by the first and last authors of this study within a one-hour workshop. Hereafter, each rater assessed five videos independently to allow for an evaluation of inter-rater reliability.

### Statistical analysis

Analysis for the SESETD was performed on each of the following categorical variables: ‘Secretion management’, ‘spontaneous swallows’, ‘laryngeal sensibility/cough’ and ‘readiness for decannulation’, as well as on a score that was built from the sum of the single items on a scale from ‘0’ (meaning no item was passed) to ‘3’ (all items passed) [12, 13, 36]. Krippendorff’s α statistics was used to determine inter-rater reliability for the single items with an α-level > 0.80 considered to show almost perfect agreement and α ≥ 0.67 allowing to draw tentative conclusions as previously described [25]. Test-retest reliability was investigated by the use of Light’s κ for the single items. As proposed by Landis and Koch, κ levels were considered as showing poor (0), slight (0.00–0.20), fair (0.21–0.40), moderate (0.41–0.60), substantial (0.61–0.80) and almost perfect agreement (0.81–1.00) [26]. In clinical practice, κ values between 0.6–1.0 are considered valid for use. For the sum score, Intraclass Correlation coefficient (ICC; 2-way random model, absolute agreement) was calculated. According to Koo and Li, values < 0.50 indicate poor, 0.50–0.75 moderate, 0.75–0.90 good and ≥ 0.90 excellent agreement [24]. All analyses were performed using SPSS Statistics 25.0 (IBM, Armonk, USA). Krippendorff’s α was calculated using the KALPHA macro, version 3.0 [18]. To provide confidence intervals for Krippendorff’s α, number of bootstrapping samples was set at 10000.

## Results

All raters completed their scoresheets without missing data. Results on α, respectively κ for the expert and non-expert group are summarized in Tables 1 and 2. With regard to the item ‘secretions’, α-levels > 0.80 in both groups indicated almost perfect agreement, as did κ in the group of experts with regard to test-retest reliability. At an α-level > 0.80, almost perfect agreement on the item ‘spontaneous swallows’ was found in both groups as well. κ indicated almost perfect agreement with regard to test-retest reliability on this item. At α-levels ≥0.67, tentative conclusions can be drawn from the evaluation of ‘laryngeal sensibility/cough’ in both groups, whereas κ indicated substantial agreement when testing test-retest reliability. With regard to this item, in both group of raters the most common misjudgment was evaluating this item as passed even though reaction to touch of the arytenoids was missing as previously defined by the first and last author of this study. Characterized by an α-level > 0.80, the item ‘decannulation’ presented almost perfect agreement among the group of experts. In the group of non-experts an α-level ≥ 0.67 allowed to draw tentative conclusions with regard to this item which was most commonly as result of misjudgment of the item ‘laryngeal sensibility/cough’ as mentioned before. Test-retest reliability showed almost perfect agreement according to κ on ‘decannulation’. To quantify inter-rater reliability on the sum score, Cronbach’s α was calculated and presented almost perfect agreement at α-levels > 0.9 for both groups. When considering the sum score in the group of experts, at an α-level of 0.92 (0.83–0.98) Cronbach’s α indicated almost perfect agreement with regard to test-retest reliability. The same sum score derived from evaluation of different items as ‘passed’ in few cases. In the group of experts, one rater evaluated ‘laryngeal sensibility/cough’ as passed whereas all other raters considered the item ‘secretions’ to be accomplished in one video. In the group of non-experts, in one video score 2 resulted from passing either ‘secretion’ and ‘spontaneous swallows’, respectively ‘spontaneous swallows’ and ‘laryngeal sensibility/cough’ in two raters.

**Table 1:** Inter-rater reliability in a group of ‘experts’ and ‘non-experts’ (*Krippendorff‘s α; **Cronbach’s α)

Item tested	α in the group of ‘experts’ (95%-confidence interval)	α in the group of ‘non-experts’ (95%-confidence interval)
Secretion	0.92 (0.84–1.00)*	0.88 (0.78–0.96)*
Spontaneous Swallows	1.00 (1.00–1.00)*	0.87 (0,78–0.96)*
Laryngeal Sensibility/Cough	0.73 (0.59–0.86)*	0.68 (0.54–0.82)*
Decannulation	0.87 (0.76–0.96)*	0.77 (0.63–0.89)*
Sum score	0.94 (0.87–0.98)**	0.91 (0.77–0.99)**

**Table 2:** Test-retest reliability in a group of ‘experts’

Item tested	Light’s κ (95%-confidence interval)
Secretion	1.0 (1.00–1.00)
Spontaneous Swallows	0.93 (0.81–1.05)
Laryngeal Sensibility/Cough	0.76 (0.41–1.11)
Decannulation	0.86 (0.64–1.09)

## Discussion

In the present study, the FEES-based SESETD has shown to be a reliable tool to evaluate decannulation readiness in critically ill neurologic patients. Of the three single items included, assessment of ‘laryngeal sensibility/cough’ has shown poorest reliability but still allowed to draw at least tentative conclusions. A sum score of the three items was introduced and showed sufficient reliability. α-levels among raters with at least 5 years of experience using the SESEDT were slightly higher compared to raters with no experience with the score on each single item as well as on the sum score and as a result from this also on the ultimate decision whether to decannulate or not.

The first item of the SESETD is ‘secretion management’. Evaluation of secretions has been included or even been the main target of several endoscopy-based swallowing scores [6, 14, 31, 33]. Increased accumulation of secretions was identified to lead to a significantly increased risk of aspiration pneumonia and respiratory distress with subsequent need for intubation and artificial ventilation [14, 43]. At an α-level > 0.80 in the present study, good reliability for assessing this feature could be confirmed which is in line with findings from former studies [6, 23, 32, 33]. Even though findings from more complex scores to evaluate secretions cannot directly be compared to the dichotomous assessment of the SESETD, it seems that the evaluation of secretions by FEES in general shows sufficient inter-rater and test-retest reliability even without prior training. It needs to be considered, however, that data on reliability were gathered from varying collectives, leading to a possible bias. As the items included in the SESETD are relevant for safe swallowing function regardless of the underlying pathology, we would expect reliability data on the single items to be comparable to a considerable degree.

The second item of the SESETD is ‘spontaneous swallows’. Spontaneous swallowing belongs to protective aerodigestive reflexes [7, 37, 38]. A reduction of spontaneous swallowing has been demonstrated as a sensitive surrogate of dysphagia in clinical populations [31]. In order to evaluate validity and reliability of the Boston Residue and Clearance Scale (BRACS), Kaneoka et al. assessed the presence of spontaneous clearing swallows as part of a 11-point ordinal scale that showed excellent inter-rater reliability and test-retest reliability [22]. With findings from the recent study that go in line with the formerly mentioned one, it seems that evaluation of spontaneous swallowing using FEES is reliable.

The third item of the SESETD is ‘laryngeal sensibility/cough’. In previous studies, two methods have been implemented to evaluate laryngeal sensibility. The so-called FEESST (= flexible endoscopic evaluation of swallowing with sensory testing), uses the reaction to an air-puff that is directed against the arytenoids provided via the working channel of an endoscope [1]. The other method uses the reaction to a light touch of the arytenoids with the tip of the endoscope (‘touch method’). Despite the touch method being limited with regard to inconsistent intensities between trials and examiners [21] Kaneoka et al. found this method to be superior to providing air-pulses for sensory testing when evaluating penetration/aspiration [19].

Using FEESST, Aviv and co-workers demonstrated pharyngolaryngeal sensory deficits to be linked to aspiration in patients post-stroke [2, 3]. Applying the touch method, laryngeal sensory loss was not only shown to be associated with compromised airway protection [19] but in dysphagic patients with an absent laryngeal adductor reflex (LAR) following touch, the risk to subsequently develop pneumonia was found to be massively increased [20]. Furthermore, Marian et al. found that the severity of laryngeal sensory loss was closely related to global swallowing impairment in patients suffering from PSD [30].

The evaluation of ‘laryngeal sensitivity/cough’ in the current investigation resulted in worse inter-rater and test-retest reliability when compared to the other two test items. This is in line with findings from a study on healthy adults in which a poor interrater agreement was found when evaluating the LAR using the touch method [21]. As stated above, in the present study all raters evaluated videos of the endoscopy with no additional information, e.g. sound or a second camera showing the patient’s reaction from the outside. Based on these findings, it is hence recommended, that when testing sensory reaction to not solely consider the endoscopic findings but also to get a clinical impression of the reaction (e.g. facial or verbal reaction). A second issue that possibly contributed to the worse reliability of the sensory testing may be seen in the specific way how this testing is carried out. Thus, after touching the arytenoids, the endoscopist usually retracts the endoscope slightly to evaluate the effect of the touch, for example a reflexive swallow. As the patient’s reaction frequently takes place immediately after the touch, it is not always easy to tell by just seeing the video whether the recorded movements were just due to movements of the endoscope or represent a true reflexive laryngeal movement indicating preserved sensory function. Consequently, it may be suggested that by adding sound and external view to the information available in this clinical scenario, reliability of this item – and as a result of the entire score – may even further be improved. If all three items were scored as “passed” patients were classified as ‘ready for decannulation’. This sum score showed excellent inter-rater reliability at a Cronbach’s α ≥ 0.90 both in the group of ‘experts’ and ‘non-experts’ as well as excellent test-retest reliability in the group of experts. If the sum score is < 3 on the first FEES after end of weaning, it might be used as a prognostic tool with higher scores presumably predicting decannulation readiness earlier than in patients scoring lower but this needs to be evaluated in further studies [36].

Instrumental evaluation of swallowing function in tendency leads to decannulation failure less often than gradual decrease of tube size and intermittent tube capping according to a recent [39]. Furthermore, in the genuine study on the SESETD patients who were assessed by clinical swallowing examination were deemed ready for decannulation significantly less often than if the assessment was based on FEES (29% vs. 54%) with a comparatively low rate of decannulation failure in 1.9% [44]. Optimal timing of decannulation still needs to be assessed further considering risks of decannulation failure with the need for reintubation/recannulation, e.g. procedure-related or early complications such as hemorrhage, pneumothorax, infection, subcutaneous emphysema or hypoxia [8, 15] compared to late complications from prolonged cannulation, such as tracheal stenosis, bleeding, fistulas, infections, aspiration as well as psychological implications and delayed rehabilitation [15, 16]. Following the relatively low risk for decannulation failure after application of the SESETD, an early evaluation of swallowing function after end of weaning should be sought to prevent from possibly unnecessary prolonged cannulation. The optimal period between follow-up FEES in patients who cannot be decannulated right away still needs to be investigated.

There are certain limitations to this study that need to be addressed. First, as mentioned above, videos did not include external view or audio signal, both of which are prone to provide information possibly relevant for clinical judgement. Second, raters in the group of ‘non-experts’ had no or only minor experience with the protocol but differed with regard to experience in using FEES. 10/27 raters had experience for more than 5 years which would likely include experience on evaluation of secretion management, spontaneous swallowing frequency and sensory testing. Only relatively small differences concerning reliability between groups may be a result of evaluation by comparatively experienced endoscopists in both groups. Conversely, our findings may not be applicable for inexperienced FEES users. The validity of the sum score has yet to be assessed for the collective of patients on the neurological ICU and findings from the recent study therefore can only be interpreted cautiously. There are indications that the sum score may aid predicting the likeliness of early decannulation from patients suffering from GBS with a high score being linked to a higher likeliness of early decannulation during the course of treatment on the ICU. Furthermore, it needs to be considered that scores 1 and 2 can derive from differing evaluations of single items by different raters. The thresholds for relevant α- and κ-levels were taken from the literature similar to other studies in the field and were not predefined particularly for this study. This may compromise with the validity of our findings. The ratio between raters and videos, particularly in the group of ‘non-experts’, may compromise with our results. In cases where the error variance between raters is much smaller than the error variance of samples, the confidence intervals increase with increasing numbers of raters [35].

## Conclusion

The SESETD-protocol includes a stepwise evaluation of ‘secretions’, ‘spontaneous swallows’, ‘laryngeal sensibility/cough’ to ultimately evaluate ‘decannulation readiness’. It demonstrates good to excellent agreement for each item in experienced and untrained raters strongly supporting its usefulness for implementation in daily clinical routine and as an outcome measure for clinical trials.

## Data Availability

The datasets used and analysed during the current study are available from the corresponding author on reasonable request.

## Abbreviations

FEES: Flexible endoscopic evaluation of swallowing
FEESST: Flexible endoscopic evaluation of swallowing with sensory testing
ICU: Intensive care unit
LAR: Laryngeal adductor reflex
OD: Oropharyngeal dysphagia
PSD: Post-stroke dysphagia
SESETD: Standardized Endoscopic Swallowing Evaluation for Tracheostomy Decannulation in Critically Ill Neurologic Patients
SLT: Speech-language pathologist
